# Supplementary material for: Content-rich biological network constructed by mining PubMed abstracts
Source: BMC Bioinformatics. 2004 Oct 8;5:147. doi: 10.1186/1471-2105-5-147 (PMC528731; doi:10.1186/1471-2105-5-147)
Supplement: Additional File 2 — The original results of the above study (non-essential files are deleted to keep the file size under the limit set by BMC bioinformatics). [file 1471-2105-5-147-S2.bz2 › chilibotAdditionalFile2/dip40/289ID1833185E1119/html/CDC2_CCNA2.html]

 


 **CDC2** and **CCNA2** 
  
Found 657 abstracts in PubMed, retrieved 40.  
 

 What does Google say? 
 PDF only 
| .edu only 

---

**Interactive relationship** (e.g. stimulation, inhibition, etc)

**Inhibitory relationship**- the phosphorylated JNK SAPK and p38 inhibited the phosphorylation of  **cdc2** , and regulated the expression of cyclin A  [ **CCNA2** ] , cyclin B1, and cdk  [ **CDC2** ]  proteins, thereby causing G2 arrest.  Ref: 12429981 Int J Oncol, 2002
- Rather, CDK  [ **CDC2** ]  inhibition was associated with a down regulation of cyclin A  [ **CCNA2** ]  and B1 expression.  Ref: 12444545 Oncogene, 2002
**Neutral relationship**- This is a novel mechanism of TGF beta1 inhibition requiring early G 1 induction and stabilization of p21 protein, which binds to and inhibits cyclin E CDK2 and cyclin A  [ **CCNA2** ]  CDK2 kinase activity rather than direct modulation of cyclin or CDK  [ **CDC2** ]  protein levels as seen in other systems.  Ref: 12810668 Cancer Res, 2003
- We now show that N terminal to the NLS domain of pro IL 16 are protein kinase CK2 substrate and  **cdc2**  kinase substrate sites which, along with the NLS, constitute a dual phosphorylation regulated CcN  [ **CCNA2** ]  motif which regulates nuclear localization of pro IL 16.  Ref: 12450396 Biochemistry, 2002

**Non-interactive relationship** (e.g. studied together, co-existance, homology, etc.)

- METHODS The molecular expressions of Rb, cyclin A  [ **CCNA2** ] , cyclin E, CDK2 and  **CDC2**  involved in S phase progression were investigated by westernblot in L 2 and Br1 3pr 1 cells.  Ref: 12600080 Hua Xi Yi Ke Da Xue Xue Bao, 2001
- The increased expression of  **Cdc2**  and cyclin A  [ **CCNA2** ]  by HKa was not associated with an apparent change in cell cycle profiles of basic fibroblast growth factor stimulated proliferating cells, but closely correlated with a marked increase of apoptosis, suggesting that the elevated  **Cdc2**  activity is involved in HKa induced apoptosis of proliferating endothelial cells.  Ref: 12742823 Am J Physiol Heart Circ Physiol, 2003
- When we examined the effects of this drug on ACHN cells, monensin decreased the levels of CDK2, CDK6,  **cdc2** , cyclin A  [ **CCNA2** ]  and cyclin B1 proteins.  Ref: 12632079 Int J Oncol, 2003
- The array data enabled us to focus on the role of potential key genes e.g., GADD153, CHK2, p21 WAF1, cyclin A  [ **CCNA2** ] , CDK1  [ **CDC2** ] , and DHFR that might be targets of MSA in impeding cell cycle progression.  Ref: 12517777 Cancer Res, 2003
- Upregulation of  **Cdc2**  and cyclin A  [ **CCNA2** ]  during apoptosis of endothelial cells induced by cleaved high molecular weight kininogen.  Ref: 12742823 Am J Physiol Heart Circ Physiol, 2003
- Progression through S phase of the eukaryotic cell cycle is regulated by the action of the cyclin dependent protein kinase  [ **CDC2** ]  2 CDK2 in association with cyclin A  [ **CCNA2** ] .  Ref: 12501191 Biochemistry, 2002
- Finally, forced expression of the NF YA protein in cells committed to differentiate leads to an impairment in the down regulation of cyclin A  [ **CCNA2** ] , cyclin B1, and cdk1  [ **CDC2** ]  expression and is accompanied by a delay in myogenin expression.  Ref: 12857858 Mol Biol Cell, 2003
- Adriamycin induced G1 S arrest seems to be realized via cyclin cdk  [ **CDC2** ]  complexes activity independent way involving antiproliferative targets downstream of cyclin E cdk2 and cyclin A  [ **CCNA2** ]  cdk2 complexes.  Ref: 12722479 Tsitologiia, 2003
- This was associated with earlier expression of the cell cycle promoting cyclin A2  [ **CCNA2** ] , cyclin E, cyclin B1, cyclin F and cyclin dependent kinase 1  [ **CDC2** ]  Cdk 1  [ **CDC2** ]  genes and diminished protein levels of Cdk  [ **CDC2** ]  inhibitor p21Cip1.  Ref: 12867420 J Biol ChemJ Biol Chem, 2003
- Increased p27 and p21 levels coincided with reduced activities of cell cycle dependent kinases cdk  [ **CDC2** ]  such as cdk2 and cyclin A  [ **CCNA2** ]  protein kinases 24 h after TRO treatment.  Ref: 12595159 Toxicol Lett, 2003
- Furthermore, cyclin A  [ **CCNA2** ]  CDK1  [ **CDC2** ]  2, cyclin B CDK1  [ **CDC2** ]  2 and cyclin E CDK2 complexes can phosphorylate multiple p73 isoforms in vitro at threonine 86 T86.  Ref: 12676926 J Biol Chem< MedlineTA>J Biol Chem, 2003
- Mitogen induced downregulation of the cyclin dependent kinase CDK  [ **CDC2** ]  inhibitor p27 kip1, and induction of the G1 cyclins cyclin D1, cyclin A  [ **CCNA2** ] , and cyclin E were also attenuated by the non thiazolidinedione partial PPARgamma agonist.  Ref: 12694805 Eur J Pharmacol, 2003
- When cells were synchronized in S phase with aphidicolin and then released, activation of Delta MEKK3 ER\* resulted in the up regulation of p21 CIP1 and a pronounced inhibition of cyclin A  [ **CCNA2** ]  CDK2 and cyclin B1 CDK1  [ **CDC2** ]  kinase activity.  Ref: 12444545 Oncogene, 2002
- Monensin decreased the levels of CDK2, CDK6,  **cdc2** , cyclin A  [ **CCNA2** ] , cyclin B1, cyclin D1 and cyclin E proteins but did not alter CDK4 protein.  Ref: 12792794 Int J Oncol, 2003
- PD treated cells were arrested at G1 S phase, which coincided well with decreases in Cyclin A  [ **CCNA2** ]  Cdk2 activity, but not in Cyclin E Cdk2 and  **Cdc2**  activities.  Ref: 12819186 Carcinogenesis< MedlineTA>Carcinogenesis, 2003
- Positive staining for cyclin D1, cyclin E, cyclin A  [ **CCNA2** ] , cyclin B1, cdk2, cdk4,  **cdc2** , p53, p21, and p27 was observed in 63%, 66%, 31%, 32%, 51%, 77%, 71%, 43%, 35%, and 60% of the 82 carcinomas, respectively.  Ref: 12792921 Hum Pathol, 2003
- Our results support an emerging hypothesis that  **Cdc2**  and cyclin A  [ **CCNA2** ]  are important regulators for cell cycle as well as for apoptosis.  Ref: 12742823 Am J Physiol Heart Circ Physiol, 2003
- We report that HKa induced a significant upregulation of  **Cdc2**  and cyclin A  [ **CCNA2** ]  in proliferating endothelial cells, concurrent with a marked increase of  **Cdc2**  activity.  Ref: 12742823 Am J Physiol Heart Circ Physiol, 2003
- p21 Waf1 Cip1 exerts a key role in driving this G 2 exit both by inhibiting cyclin B1 Cdk1  [ **CDC2** ]  and cyclin A  [ **CCNA2** ]  Cdk1  [ **CDC2** ]  2 complexes, which control G 2 M progression, and by blocking the phosphorylation of pRb family proteins.  Ref: 12881433 EMBO JEMBO J, 2003
- Western blotting analysis revealed that the protein levels of cyclin A  [ **CCNA2** ] , cyclin E, and cyclin dependent kinase CDK  [ **CDC2** ]  2 but not cyclin D1 and CDK4 decreased after progesterone treatment, but those of CDK  [ **CDC2** ]  inhibitory proteins, p21 and p27, increased.  Ref: 12810531 Endocrinology< MedlineTA>Endocrinology, 2003
- Treatment with STI571 also led to reduction of cyclin A  [ **CCNA2** ] , B1, and  **CDC2**  levels.  Ref: 12679488 J Clin Endocrinol Metab, 2003
- We identified 10 suppressors classified into three groups 1 gene products regulating Cdk1  [ **CDC2** ]  activities, cdk1  [ **CDC2** ]  and cyclin A  [ **CCNA2** ] .  Ref: 12454065 Genetics, 2002
- However, in both cell types Cdk  [ **CDC2** ]  2 activity associated with cyclin E and cyclin A  [ **CCNA2** ]  showed an increase only when the cells transited from G 1 into S phase.  Ref: 12704643 J Cell Physiol, 2003
- The DNA histogram showed that the cell fraction of the G2 M phase was increased, and this G2 M phase arrest was related to a decrease of cdk1  [ **CDC2** ]  and cyclin A  [ **CCNA2** ] .  Ref: 12679801 Cancer Gene Ther, 2003
